# Quantifications of Oleocolloid Matrices Made of Whey Protein and Oleogels

**DOI:** 10.3390/foods9111697

**Published:** 2020-11-19

**Authors:** Clifford Park, Rafael Jimenez-Flores, Farnaz Maleky

**Affiliations:** Department of Food Science and Technology, The Ohio State University, 2015 Fyffe Ct., Columbus, OH 43210, USA; park.1747@osu.edu (C.P.); jimenez-flores.1@osu.edu (R.J.-F.)

**Keywords:** oleocolloid, hydro-oleocolloid, protein denaturation, hydrophobic interactions, structure, dispersion and solubilization

## Abstract

Consumer demand for high protein content and plant-based fat has necessitated novel approaches to healthy food products. In response to this need, oleogels (OG) (structured liquid oils) emerged as a possible means of not only replacing saturated and trans fats but also delivering food protein. Nevertheless, an in-depth view of the structure of networks made of OG and protein is deficient. Hence, the objective of this study is developing oleocolloid (OC) (whey protein and rice bran wax OG) and hydro-oleocolloid (HOC) (OC + water) matrices with varying protein content (2.5–7.5%) to characterize their structural properties. Thermal analysis of the matrices via differential scanning calorimetry (DSC) documented the effects of hydrophobic interactions on the protein structure and its stability. Whey protein denaturation temperature increased from 74.9 °C to 102.8 °C in the presence of high oleic soybean oil. The effects of vegetable oil on WPI structure was also verified by FTIR spectroscopy. Data analysis revealed slight structural changes of the WPI secondary structure in the hydrophobic oil medium and the α-helix and β-sheet proportion in the emulsion medium was significantly altered. Similar analysis was performed in OC and HOC networks to quantify possible interactions between protein and rice bran wax. Results indicated that the protein was denatured during the thermal and mechanical conditions required for the oleogelation process, while it did not affect the systems’ solid fat content (SFC) and polymorphic patterns of the oleogels. However, DSC analysis showed different onset of melting for OC and HOC samples due to colloidal interactions between the protein and the lipid phase. The role of these chemistry was confirmed by microscopy analyses where OC and HOC matrices displayed notably different microstructural properties. The observed differences in the structural properties between OC and HOC matrices indicate the different colloidal interactions mediated by oleogelation process and the liquid medium type (oil vs. emulsion).

## 1. Introduction

Over the past decades, oleogelation technology has emerged as a promising technology to replace hardstock fats. Oleogelation involves a process of creating semi-solid lipid systems called oleogels (OG) with organic liquids and organogelators [1,2]. Numerous OGs have been formed with different combinations of liquid oils and organogelators in order to mimic textural attributes of saturated fats [3,4,5]. In fact, many studies have incorporated OGs in food products including cookie, sausage, ice cream, frankfurters, and cream cheese [6,7,8,9,10,11]. These studies have documented that OGs could not only provide similar textural attributes to saturated fats but also enhance nutritional profiles [6,8,9]. For example, Bemer et al. [11] reported that a cream cheese product made with rice bran wax OG had a healthier fatty acid profile with about 25% reduction in its total fat content and about 120% increase in polyunsaturated fatty acids content [11]. Thus, OGs appear to be promising alternative options for not only the reduction and/or elimination of saturated and trans fats but also the textural development in food products. 

Despite significant advances in the oleogelation technology, there is still lack of an in-depth understanding of how OGs interact with non-fat macromolecules such as proteins. Previously, colloidal gels have been developed with food proteins. For example, de Vries et al. [12] developed whey protein OGs by first creating whey protein aggregates from thermal treatment and then using a solvent exchange approach to replace water with oil [12]. In their studies, whey protein aggregates were able to generate a viscoelastic network similar to protein gels in water. In addition, Tavernier et al. [13] formed an OG network utilizing a combination of soy protein and κ-carrageenan, demonstrating that proteins and polysaccharides can be utilized to structure the hydrophobic liquid oils [13]. In these studies, food protein was thought to be involved in stabilizing the colloidal network by both covalent and non-covalent interactions [12,13]. Nevertheless, it still remains unclear how proteins are involved in hydrophobic oil-based systems. In particular, the effects of protein–lipid interactions on structural properties of these colloidal networks are not quite understood.

This knowledge gap in how protein behaves in oils is due to the fact that water is the most favorable medium for protein folding/unfolding [14,15]. Unlike non-conductive liquid oils, there are electrostatic and ionic interactions between water molecules and proteins that play crucial roles on protein aggregation and gelation [14,16]. Specifically, these interactions between water and proteins are the driving forces for proteins to form a three-dimensional network in which water is entrapped. Moreover, hydrogen bonding provided from water is known to facilitate structural conformational changes of protein such as the transformation between α-helices and β-sheets [17]. For these reasons, the gelation capacity of proteins in numerous hydrogels and emulsions has been extensively reviewed in aqueous solutions or mixtures of liquids containing water for various applications [18,19,20]. Even though water is largely involved in protein structure, it is important to note that hydrophobic proteins or inner cores of proteins can still interact with oil and consequently stabilize interfaces of proteins and oil [21]. Nevertheless, it still remains uncertain how protein structural conformational changes—which are responsible for gelation and therefore texture—occur in strict hydrophobic environment. To investigate how proteins and oleogels interact within the hydrophobic environment and form three-dimensional structures, two colloidal matrices called oleocolloid (OC) and hydro-oleocolloid (HOC) were developed with whey protein, and their structures were examined. 

## 2. Materials and Methods

### 2.1. Materials

Whey protein isolate (WPI) (93.0% *w*/*w* protein, 0.1% *w*/*w* lactose, 1.3% *w*/*w* fat, 4.7% *w*/*w* water, and 2.7% *w*/*w* ash), high-oleic soybean oil (HOSO) (8% C16, 5% C:18, 80% C18:1, and 7% C18:2), and rice bran wax (RBW) (long fatty chain esters of fatty acids (C16~C32) and fatty alcohols (C24~C38), with the major components being C22 and C24 fatty acids and C30 fatty alcohols) [22,23] were generously supplied by Hilmar Ingredients (Hilmar, CA, USA), Mallet and Company Inc (Carnegie, PA, USA), and Koster Keunen (Watertown, CT, USA), respectively. Nile Red and Fast Green FCF dyes were purchased from Fisher Scientific (Waltham, MA, USA).

### 2.2. Oleocolloid (OC) and Hydro-Oleocolloid (HOC) Processing

Both OC and HOC were prepared with three different WPI concentrations (2.5%, 5%, and 7.5% *w*/*w*) and two different ratios of HOSO and RBW (99:1 and 90:10 *w*/*w*). In this study, colloidal gels prepared with the former and the latter ratios are referred as 1 and 10% RBW systems. Exact concentrations of ingredients for OC and HOC preparations are reported in Table 1. 

For the OC formulation, WPI, HOSO, and RBW were placed in a cell connected to a water bath held at 90 °C (Polyscience, Niles, IL, USA). These materials were mixed at 260 s^−1^ using a Caframo stand mixer (Georgian Bluffs, ON, Canada). Once the temperature of the mixture reached 85 °C, an additional mixing was applied for 5 min to remove lipid crystal history. The OC samples were then stored at 5 °C (to induce oleogelation or crystallization of oil-wax mixture) for 24 h prior to performing analytical measurements. For the HOC samples, WPI and distilled water were first placed in a shearing cell kept at 50 °C and mixed at 260 s^−1^ for 10 min. Then, the fat phase (HOSO and RBW) were added, followed by ramping up the temperature of the shearing cell to 90 °C while continuously mixing the ingredients. Similar to the OC preparations, the HOC samples were mixed for an additional 5 min once at 85 °C. The HOC samples were stored at 5 °C for 24 h before performing analyses. 

To evaluate various effects of whey protein–lipid interactions on the structural properties of the OC and HOC samples, two sets of control groups were prepared. First, a control group without RBW was formulated. Colloidal dispersions (CD) (WPI and HOSO) and water in oil (W/O) colloidal emulsions (CE) (WPI, distilled water, and HOSO) were prepared at three different WPI concentrations (2.5%, 5%, and 7.5% *w*/*w*). In these colloidal systems, concentrations of HOSO were increased by the amount of RBW required for the OC and HOC networks. Second, a control group without WPI was prepared. Specifically, OG made of HOSO and RBW and hydro-oleogel (HOG) consisting of distilled water, HOSO, and RBW were prepared with 1 and 10% RBW as shown in Table 1. The CDs and OG were formulated using the OC sample preparation procedure while the CEs and HOG were formed with the HOC sample preparation method. The control samples were stored at 5 °C for 24 h before performing analyses.

### 2.3. Differential Scanning Calorimetry (DSC)

First, the potential effects of thermal and mechanical processing involved in the OC and HOC sample preparations on whey protein denaturation were analyzed using a differential scanning calorimeter (DSC Q 2000, TA Instruments, New Castle, DE, USA). To do so, 5% WPI (*w*/*w*) solution (in distilled water), 5% WPI (*w*/*w*) CD (in HOSO), and 5% WPI (*w*/*w*) CE (in distilled water and HOSO) samples were prepared by mixing gently at room temperature for 10 min. After mixing, about 10~20 mg of the samples were packed hermetically in aluminum pans. Reference pans for the WPI solution, dispersion, and emulsion samples were prepared with distilled water, HOSO, and distilled water/HOSO mixture, respectively. These samples were then placed in a sample chamber and heated from 40 to 120 °C at a heating rate of 1 °C/min. Protein denaturation temperatures in water, oil, and emulsion were acquired. Then, the CDs and CEs prepared from the OC and HOC processing (involving heating at 90 °C and shearing at 260 s^−1^) were also subject to the same heating profile in order to determine whether the OC and HOC processing denatured the whey protein. 

Second, potential whey protein–lipid interactions as well as roles of whey protein on lipid crystallization growth were investigated by examining thermodynamics of the colloidal networks processed with oleogelation in the presence of rice bran wax. Thermal properties including enthalpy (ΔH, J/g), onset of melting (°C), and melting peak (°C) of the OC and HOC samples were compared with those of the OG and HOG (the control group without protein). An empty aluminum pan was used as a reference and a heating rate of 1 °C from 10 to 90 °C was applied. For all DSC analyses, the calibration was achieved with an indium standard with a melting point of 156.6 °C and enthalpy value of 28.45 J/g. Temperature ranges of thermal events (onset and offset) were determined by a method provided by Bouzidi et al. [24]. Specifically, temperatures at which the baseline of thermograms deviated significantly were recorded using first derivatives and marked as onset and offset of thermal events. Using these onset and offset temperatures, ΔH and MP were analyzed using a TA Universal Analysis 2000 software (TA Instruments, New Castle, DE, USA). DSC experiments were performed in triplicates for all samples. 

### 2.4. Fourier Transform Infrared (FTIR) Spectroscopy 

In addition to the DSC experiments, FTIR spectroscopy was used to examine effects of the OC and HOC processing on the whey protein structure in the CD and CE samples. Only these samples (prepared in the absence of wax) were tested due to large peaks of rice bran wax functional groups that overlapped with those of protein structures of interest. Specifically, CDs and CEs prior to the OC and HOC processing and post processing were compared to highlight changes in whey protein structure. All IR spectra in the range from 3500 to 1000 cm^−1^ at a spectrum resolution of 4 cm^−1^ and with 256 scans were obtained using a FTIR instrument, Renishaw–Smiths Detection Combined Raman-IR Microscope (Reinshaw, Wotto-under-Edge, UK) occupied with a diamond attenuated total reflectance accessory. Data analysis first involved spectral subtractions of non-protein ingredients so that spectra with only protein components remained. Then, deconvolution and normalization of FTIR spectra region of 1700–1600 cm^−1^ were applied using a Peakfit v. 4.12 software (Systat Software Inc., San Jose, CA, USA). This region represents the amide I region, which was chosen to assess the secondary structure of whey protein. Then, relative % of IR bands was measured by calculating area of each curve. The spectral acquisitions were performed in triplicate for all samples. 

### 2.5. Solid Fat Content (SFC) via Pulsed Nuclear Magnetic Resonance (NMR)

In addition to DSC, potential roles of whey protein on lipid crystallization were also studied by measuring the amount of SFC in the OC and HOC matrices and comparing them with those of the OG and HOG, respectively. SFC was measured with a Bruker Minispec MQ 20 NMR machine (Billerica, MA, USA). Specifically, a direct SFC method was utilized with an f-factor of 1.5. To accurately determine SFC of the OC and HOC networks consisting of nonfat materials, false contributions of nonfat materials to SFC measurements were quantified. To quantify the measurements, the apparent SFC values of the OC and HOC samples were first measured. Then, solid fats within these samples were molten at 80 °C, knowing that OGs made with RBW have melting points ranging from 65 to 73 °C [22,25]. Then, SFC of the developed gels were calculated by subtracting SFC of nonfat materials (SFC readings of the molten samples) from the apparent SFC of samples. A total of nine SFC measurements were recorded and averaged for each sample. 

### 2.6. X-ray Diffractometry (XRD) 

To investigate possible effects of protein-particle interactions on lipid crystallization, wide angle diffraction patterns of the developed colloidal gels processed with oleogelation (in the presence of wax) were analyzed using a Rigaku MiniFlex 600 X-ray Diffractometer (Rigaku, Tokyo, Japan) equipped with a Cu source and a voltage capacity of 40 kV and 15 mA. Samples were placed in glass sample holders with 0.5 mm depth and stored at 5 °C for 24 h prior to measurements. Then, angular scans (2 θ) were performed from 5 to 30° at a scanning rate of 1° 2 θ/min at room temperature. XRD measurements were performed in triplicate. 

### 2.7. Confocal Microscopy

Microstructural properties of the whey protein colloidal networks were studied with a Leica TCS SL confocal microscope equipped with six different laser lines (Leica Microsystems, Wetzlar, Germany). Both Nile Red (0.005% *w*/*w* in acetone) and Fast Green FCF (0.01% *w*/*w* in distilled water) dyes were utilized to stain the lipid and protein components, respectively. About 10 μL of each dye solution was applied to 100 mg of the samples, and the stained samples were placed on microscope slides. To visualize both lipid and protein components, two laser lines with excitation wavelengths of 543 and 633 nm were utilized, respectively. Three confocal images were obtained at 10× magnification for each sample. Confocal images were used to quantify mean particle area (μm^2^) and percent filled area (%) of protein networks within the OC and HOC matrices. First, images were threshold adjusted. Then, a particle size analysis function was used using an ImageJ software (National Institute of Health, Bethesda, MD, USA). 

### 2.8. Polarized Light Microscopy (PLM)

Lipid crystal networks of the OG, HOG, OC, and HOC networks that were processed with oleogelation in the presence of RBW were examined using a Carl Zeiss Axio Imager 2 PLM microscope equipped with a MRc 5 camera (Carl Zeiss Microscopy, Jena, Germany). Lipid microstructures of these samples were observed at room temperature after 24 h of storage at 5 °C. PLM images were obtained at 10× and 50× magnifications. Images were processed to calculate mean particle area and percent filled area (%) of lipid crystal networks within these samples in the same manner as described above in Confocal Microscopy section.

### 2.9. Statistical Analysis

Analysis of variance (ANOVA) testing with a Tukey’s post hoc analysis was used to determine the statistical significance of the obtained data using a Prism 8 software (GraphPad, La Jolla, CA, USA). The statistical analysis was performed at the significance level of *p* < 0.05. 

## 3. Results and Discussion

**Figure 1:**
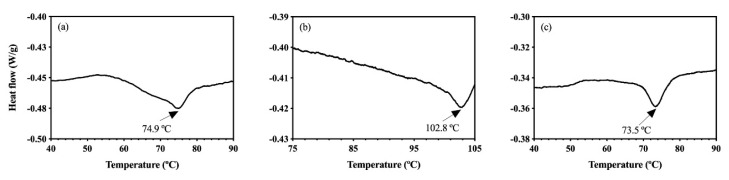
Thermal denaturation profiles of: (**a**) the 5% whey protein (*w*/*w*) in distilled water solution, (**b**) CD made with 5% whey protein (*w*/*w*), and (**c**) CE made with 5% whey protein (*w*/*w*). Arrows show the whey protein denaturation temperature.

Considering the thermal and mechanical applications in OC and HOC processing and their effects on whey protein structure, it was important to first investigate the effects of these processing conditions in the absence of oleogelation. To begin, different whey protein isolate matrices including a solution (WPI in distilled water), dispersion (WPI in oil), and emulsion (WPI in distilled water + oil) were made and analyzed through DSC. Whey protein solution, depicted in Figure 1a, showed whey protein denaturation temperature at 74.9 °C, which agrees with the findings of Boye et al. [26]. Boye et al. reported a range of denaturation temperatures for β-lactoglobulin (from 72.3 to 82.7 °C) depending on the pH of aqueous solution [26]. 

As shown in Figure 1b, the denaturation temperature of the WPI in the CD in the presence of hydrophobic oil (102.8 °C) was significantly higher than the whey protein solution (74.9 °C). Previously, it has been understood that both protein folding and unfolding need water as a solvent, since water is thermodynamically favorable for movements of amino acid backbone and side groups through hydrogen bonds [15,27]. However, the presence of moisture (about 4.7% *w*/*w*) in the WPI used in this study could induce the protein denaturation or unfolding process in the hydrophobic oil. Studies have shown that the higher denaturation temperature represents an enhanced thermal stability of globular proteins [28,29,30]. Consequently, the observed whey protein denaturation around 100 °C indicated the effects of hydrophobic interactions (protein–protein and/or protein–lipid) in the CD sample, resulting in the enhanced thermal stability or stabilization of the whey protein structure [31,32]. This result may suggest that the whey protein undergoes the denaturation or structural conformational changes differently in oil compared to water, and such structural transitions are heavily affected by degrees of hydrophobicity of liquid medium (oil vs. water). In contrast, as shown in Figure 1c, the protein denaturation temperature of the CE is 73.5 °C, similar to that of the WPI solution (74.9 °C). This result implied that the WPI CE may not have a high stabilization based on the lower denaturation temperature in this system. One possible explanation could be the solubilization of protein mainly in the water phase, which favors the water–protein interaction rather than the lipid–protein one. Such a result indicates that the entropically driven interaction between protein and water is significant even with the small molality of water in the system. This interaction is possible considering the hydrophilic nature of the whey protein surface in its native form and the strong incompatibility between water and oil [33,34].

These results also highlight that the order of liquid solvent addition could result in different chemical interactions and possibly affect the physical properties of the final products. In the CE sample, the whey protein was dissolved in water first before adding oil. This process induced the whey protein interaction with water phase, which helps explain the similar whey protein denaturation temperatures of the WPI solution and emulsion. If water had been added after dispersing the protein in oil, it was speculated that the whey protein would have different structural arrangements and exhibit different interactions similar to the CD system (Figure 1b). Data shown in Figure 1 document that the WPI used in this study was in its native structural forms and was a suitable matrix for quantifying the effects of oleogelation processing on the protein denaturation. 

**Figure 2:**
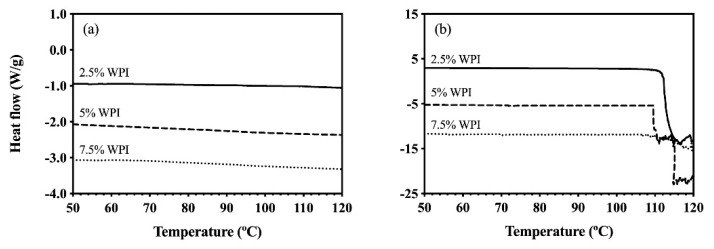
DSC thermograms of the samples with different protein concentrations: (**a**) CD samples and (**b**) CE samples. Samples were processed under the thermal and mechanical conditions used for OC and HOC, respectively. The straight and dotted lines represent samples made with different WPI concentrations.

To examine the effects of oleogelation processing on the protein structure, the thermal and mechanical applications required for OC and HOC samples were applied to the CD and CE (OC and HOC formulations with 0% rice bran wax, respectively). As illustrated in Figure 2a, the DSC thermograms of the CDs showed no protein denaturation peak when heated to 120 °C. These results were consistent for all whey protein concentrations (2.5%, 5%, and 7.5% *w*/*w*), documenting the whey protein denaturation during the OC processing (shearing and heating at 90 °C). 

Considering the protein denaturation temperature in HOSO without oleogelation processing (102.8 °C, shown in Figure 1b), Figure 2a illustrates the significant effects of oleogelation process on the WPI denaturation. The effects of the combination of thermal and mechanical energy applied for oleogelation were greater than the thermal processing alone and required less heat for the whey protein denaturation. This result is comparable with Manoi and Rizvi [35] and Simmons et al. [36] who reported whey protein denaturation was accelerated when sheared and thermally treated simultaneously [35,36]. Similar effects of oleogelation processing were seen for the CE samples as shown in Figure 2b. No noticeable endothermic peak is seen around 73.5 °C, the observed denaturation temperature of the CE prepared without the HOC processing. A closer look at Figure 2b shows sudden changes in the thermogram’s baseline around 110 °C, representing the transition of water to gas and the disruptions of the hermetically sealed DSC pans. 

**Figure 3:**
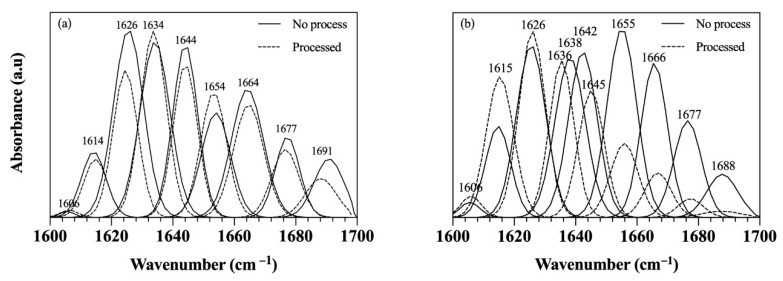
FTIR spectra of: (**a**) CD made with 5% whey protein (*w*/*w*) and (**b**) CE made with 5% whey protein (*w*/*w*), prior to and post the OC and HOC processing. Straight and dotted lines show the FTIR spectra of the samples before and after processing, respectively.

The extent of protein structural changes from the processing was assessed next using FTIR spectroscopy. As depicted in the FTIR spectra in Figure 3a, for samples with 5% protein, the CD samples prepared prior to (the straight line) and post the OC processing (the dotted line) contained various FTIR bands of whey protein within the amide I region, including an amino acid side chain peak around 1606~1608 cm^−1^, as well as peaks of β-sheet structures ranging from 1615~1638 cm^−1^ and 1677~1691 cm^−1^ [37,38]. In addition, FTIR bands of random coils, α-helix, and β-turns were observed around 1644, 1654, and 1665 cm^−1^, respectively [37,38,39]. Similar observations were seen for the CD samples with 2.5 and 7.5% (*w*/*w*) protein. Interestingly, the FTIR spectra of the CD sample processed with the OC processing was quite similar to that of the one without the processing (Figure 3a), highlighting slight structural changes after the OC processing. This is in agreement with the findings from DSC where the whey protein exhibited the enhanced thermal and structural stability due to its hydrophobic interactions with the oil medium (Figure 1b). 

To better explain the observed finding, relative % of the individual FTIR bands were quantified and reported in Table 2. As shown in Table 2, the most abundant secondary structure of whey protein was β-sheet (51.0~58.2%), followed by α-helix (13.0~19.5%), random coils (14.4~18.4%), β-turns (7.84~13.8%), and amino acid side chains (0.66~1.38%). The observed distribution of the whey protein secondary structures was consistent to whey protein isolate [38,40], and relative % of these FTIR bands in the CDs was shown to be comparable even after the OC processing. While these results confirm the insignificant structural changes due to the thermal and mechanical applications during the OC processing (Table 2), the effects of hydrophobicity on the whey protein structure are highlighted [41]. Few expectations observed for the higher protein concentration. For instance, relative % of amino acid side chains in the sample containing 7.5% (*w*/*w*) protein was significantly reduced from 1.38% to 0.66% after the OC processing while those prepared with 2.5% and 5% (*w*/*w*) protein were consistent (Table 2). In addition, relative % of α-helix in the CD sample made with 7.5% (*w*/*w*) protein increased significantly after the processing (16.2% to 19.5%) while that of the sample containing 2.5% (*w*/*w*) protein decreased after the OC processing (18.6% to 13.0%) (Table 2). These few inconsistent results could be related to the varying proportions of whey protein structures or different ratios of monomer–dimer phases of whey protein. Similar observation is reported by Lefèvre and Subirade [42,43] who noted slightly different FTIR spectra of β-lactoglobulin solutions as a function of increasing β-lactoglobulin concentrations. 

In contrast, Figure 3b demonstrates considerably different whey protein structures for the CEs processed with the HOC processing (shown in dotted line) compared to those made without the processing (shown in straight line). Comparing Figure 3a,b (reported in Table 3) shows that the whey protein denaturation or structural transitions were more significant in the presence of water. Among the five secondary structures of whey protein, β-sheet, and α-helix were the major structures affected by the HOC processing. Specifically, relative % of β-sheet in the CE samples increased significantly while that of α-helix decreased drastically for all whey protein concentrations (Table 3). These results were consistent with other studies that investigated the effects of thermal treatments on the secondary structural changes of whey protein [44,45,46]. In particular, Ngarize et al. [46] showed that the whey protein denaturation mechanism involves a loss of helical structures (α-helix) and formation of stable intermolecular β-sheets that are responsible for a gel formation [46]. While no consistent result is seen for amino acid side chains in the CE samples, after the HOC processing, random coils and β-turns show increasing and decreasing patterns, respectively, (Table 3). Extent of random coils was expected to increase due to an elevated extent of disorganization of protein structures during the protein denaturation or unfolding process. This increase in the random coil content is thought to be an entropically gain, which drives an irreversible aggregation [44,45]. The observed slight decrease in relative % of β-turns was also expected as the formation of β-sheets takes place after losses of β-turns and unordered structures of whey protein [44,47]. Overall, the obtained results from DSC and FTIR analyses thus far highlight the different colloidal chemistry induced by the types of liquid medium (oil vs. emulsion), which may affect physical properties of the developed colloidal gels.

**Figure 4:**
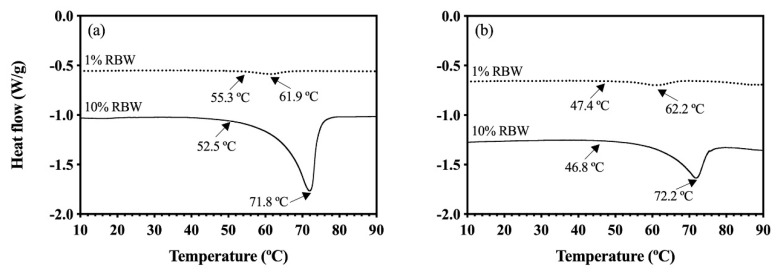
DSC melting thermograms of: (**a**) OG and (**b**) HOG made with 1 and 10% RBW. Arrows show the onset of melting and melting peak. The straight and dotted lines represent samples made with different RBW concentrations.

To further investigate the effects of these processing conditions and the whey protein interactions with rice bran wax, further DSC analyses were performed on the OC and HOC samples prepared with rice bran wax. The whey protein denaturation during the colloidal network formation can expose its hydrophobic cores to lipids that may affect lipid crystallization properties. This exposure may be better explained by considering β-lactoglobulin, a lipocalin protein with a high affinity for fatty acids and triacylglycerides [48,49]. To better investigate the impacts of the whey protein denaturation on the lipids network, the crystallization of the OG and HOG in the absence of whey protein were first investigated. Thermal properties including onset of melting and melting point of the OG and HOG are shown in Figure 4a,b, respectively, as well as Table 4. 

As depicted in Figure 4b, the onset of melting of the HOG samples made of 1% RBW was 47.4 °C, which was lower than that of the OG made with 1% RBW (55.3 °C) (Figure 4a). Similarly, the HOG sample made with 10% RBW showed the lower onset of melting (46.8 °C) (Figure 4b) compared to that of the 10% RBW OG (52.5 °C) (Figure 4a). These findings indicated that the presence of water in the HOG influenced the lipid crystallization.

It is important to note that RBW consists of long fatty chain esters of fatty acids (C16~C32) and fatty alcohols (C24~C38), with the major components being C22 and C24 fatty acids and C30 fatty alcohols [22,23]. In addition, HOSO contains about 0.1% of free fatty acids and 1.7% polar compounds [50]. This composition is important to note because hydroxyl groups of fatty alcohols from RBW and free fatty acids and polar compounds within oil may be capable of forming hydrogen bonds with water [51,52]. Then, such hydrogen bonds between water and lipid materials may have partially impeded the oleogel crystallization process within the HOG sample, thus lowering its onset of melting. Even though the start of melting between the HOG and OG differed, the melting points of the OG and HOG were comparable with respect to their RBW levels (around 62.0 °C and 72.0 °C for 1 and 10% RBW, respectively) as illustrated in Figure 4.

**Figure 5:**
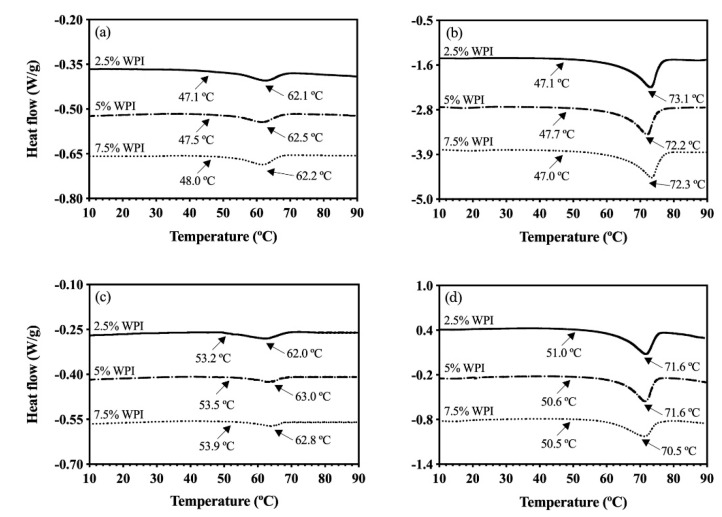
DSC melting thermograms of: (**a**) OC samples gelled with 1% RBW, (**b**) OC samples gelled with 10% RBW, (**c**) HOC samples gelled with 1% RBW, and (**d**) HOC samples gelled with 10% RBW. Arrows show the onset of melting and melting peak. The straight and dotted lines represent samples made with different WPI concentrations.

The same melting points between the OG and HOG despite the latter containing water suggested that the bulk oleogel crystal networks between these OGs were similar. The observed higher melting points of the 10% RBW OG and HOG compared to those made with 1% RBW was consistent with other works that previously demonstrated that melting points of wax OGs are directly related with wax concentration [22,53]. Specifically, increasing wax content has been shown to result in a greater supersaturation, subsequently generating a greater driving force for the lipid crystallization [53]. Similar to the HOG system, the OC samples displayed significantly lower onset of melting around 47.0 °C regardless of protein and wax concentrations (Figure 5a,b) compared to the OG (Figure 4a). 

The lower onset of melting of the OCs indicated that whey protein affected the melting behavior of the oleogel crystals presumably due to the heterogeneity effects, which are known to affect thermal properties [54]. Even though the onset of melting of the OCs was significantly lower than the OGs, the melting points of the 1% RBW OCs were around 62.0 °C (Figure 5a), which was comparable to that of the 1% RBW OG sample (61.9 °C, Figure 4a). The same observation was documented for the 10% RBW OCs whose melting points were around 72.0 °C (Figure 5b) matching that of the 10% RBW OG (71.8 °C) (Figure 4a). These findings illustrated that the melting properties of oleogel crystal networks were not too significantly influenced by the presence of whey protein. The start of melting for the HOCs made with 1% RBW ranged from 53.2–53.9 °C (Figure 5c), higher than that of the 1% RBW HOG (47.4 °C) and similar to that of the 1% RBW OG (55.3 °C) as reported in Table 4 and depicted in Figure 4.

The consistent pattern was seen for the onset of melting for the HOCs made with 10% RBW. Specifically, the 10% RBW HOCs started melting around 50.5–51.0 °C (Figure 5d) while the onset of melting for the 10% RBW HOG and OG was 46.8 °C and 52.5 °C, respectively (Figure 4). Theoretically, the greater extent of heterogeneity (in terms of numbers of ingredients and their concentration variations) affects thermal properties of a given network [54]. Nevertheless, both onset of melting and melting peak of the HOCs (regardless of protein and wax content) were consistent with the OG control (Table 4), highlighting that the combination of protein and water did not largely affect the thermal properties of oleogel crystal networks. This finding could be explained by the entropically driven interaction between protein and water, strongly bonding with water rather than with oil, as previously discussed regarding the significantly different WPI denaturation temperatures (Figure 1b,c) and the protein structural changes (Figure 3). The observed discrepancies in the network melting profiles highlighted the effects of different protein chemistry mediated by liquid solvent (hydrophobic oil vs. emulsion) on their thermal properties. 

In order to further investigate the effects of the interactions between whey protein and solvent types on the oleogelation process within the colloidal networks, enthalpy (ΔH) values of the endothermic melting events from DSC as well as solid fat content (SFC) of these colloidal networks determined through pulsed NMR were quantified. Given the achieved whey protein denaturation during the OC and HOC processing (Figure 2), the obtained ΔH values were attributed to the samples’ thermal entities other than the protein denaturation. These entities may include melting of oleogel crystal networks and possible interactions among constituents of the colloidal networks. Specifically, ΔH of the 1% RBW OG was 0.61 J/g while that of the 1% RBW OCs significantly decreased with more whey protein concentrations from 0.65 to 0.38 J/g in the OCs with 2.5% and 7.5% (*w*/*w*) protein, respectively, as shown in Table 4. The obtained ΔH values of these samples correlated with their SFC. In particular, the SFC of 1% RBW OG sample was 0.76% while the SFC of the OCs containing 2.5% and 7.5% (*w*/*w*) protein dropped from 0.73% to 0.63%, respectively (Table 4). The 10% RBW OG and OC samples showed the consistent trend where both ΔH and SFC decreased as a function of increasing protein content. For example, the ΔH and SFC of the 10% RBW OG were 19.8 J/g and 8.75%, respectively. When whey protein content was increased from 2.5% to 7.5% (*w*/*w*) in the 10% RBW OCs, lower ΔH (20.1 to 17.1 J/g) and SFC (8.35% to 7.82%) were obtained (Table 4). These results indicated that both ΔH and SFC values of the OG and OC systems showed a linear relationship with the amount of rice bran wax. This result was an expected outcome as more wax would result in a greater solid fat formation as Martini et al. [53] previously demonstrated a direct relationship between wax content and ΔH of lipid crystal melting in sunflower oil OGs made with paraffin and bees waxes at concentrations ranging from 1% to 10% [53]. This consistent trend was observed for the HOGs and HOCs. Particularly, the ΔH and SFC of the 1% RBW HOG sample were 0.60 J/g and 0.60% while those of the 1% RBW HOCs decreased from 0.41 to 0.21 J/g and 0.59 to 0.47% SFC with increasing protein content (Table 4). Likewise, both ΔH and SFC of the 10% HOCs decreased with more whey protein as shown in Table 4. It is important to note that the HOG and certain HOCs showed comparable ΔH with the OG and OCs despite having less SFC in their networks (Table 4). For example, the ΔH of the 1% RBW HOG (0.60 J/g) was equivalent with that of the 1% RBW OG (0.61 J/g). Additionally, the 1% RBW HOCs made with 5 and 7.5% (*w*/*w*) whey protein showed comparable ΔH (0.27 and 0.21 J/g) with the 1% RBW OC containing 7.5% protein (0.38 J/g). Similar observations were documented for the 10% wax systems. Specifically, the ΔH of the 10% RBW HOG and OG were 20.1 and 19.8 J/g, respectively. In addition, the ΔH of the 10% RBW HOC made with 2.5% (*w*/*w*) protein was 18.6 (J/g), which was similar to those of all OCs (17.1–20.4 J/g). The 10% RBW HOC with 5% protein (*w*/*w*) also showed the comparable ΔH (17.1 J/g) with the 10% RBW OC made with 7.5% (*w*/*w*) protein (17.1 J/g). 

These results confirmed that the ΔH of the HOG and HOC entailed not only the melting of the oleogel crystals but also energies associated with breaking chemical intermolecular bonds such as hydrogen bonds between water and non-polar lipids [55]. This is in agreement with Bennett et al. [55] who noted the hydrogen bonds between water and lipid materials (1,2-dilauroyl-sn-glycero-3-phosphocholine and 1,2-dimyristoyl-sn-glycero-3-phosphocholine) led to an increase in enthalpy. Their observations were reasonable as breaking a single hydrogen bond in liquid water and around non-polar substances is thermodynamically significant [56,57]. Hence, the obtained findings verify that the ΔH of the HOC systems was increased due to hydrogen bonds between water and oil and wax components present in the systems. The acquired SFC values were also used to assess the possible role of whey protein on the lipid crystallization growth. To more accurately assess whether the whey protein promoted the lipid crystal growth within the OC and HOC networks, the SFC of these matrices were normalized to 100% lipid content of HOSO and RBW and reported in Table 4 as SFC*. As shown in that table, the SFC* of the 1% RBW networks were consistent, ranging from 0.66 to 0.79% except for the 7.5% WPI (*w*/*w*) HOC sample (0.66% SFC*). The significantly lower SFC* from this sample could be due to the heterogeneity effects. Specifically, this particular sample had the greatest extent of ingredient concentration variations, and such a heterogenous nature is known to be capable of affecting crystal growth processes leading to structural heterogeneity, crystal imperfections, surface pores, dislocation sites, and so on [58]. The comparable SFC* values were also obtained for the 10% RBW systems (except for the HOC containing 7.5% (*w*/*w*) protein due to the heterogeneity effects) as reported in Table 4. 

**Figure 6:**
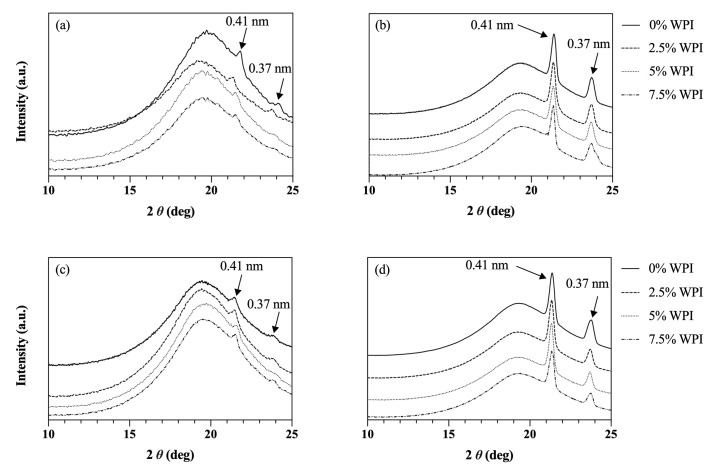
XRD spectra of the colloidal gels prepared with varying WPI and RBW content: (**a**) the OG (0% WPI) and OCs made with 1% RBW, (**b**) the OG (0% WPI) and OCs made with 10% RBW, (**c**) the HOG (0% WPI) and HOCs made with 1% RBW, and (**d**) the HOG (0% WPI) and HOCs made with 10% RBW.

Considering the objective of this study and the possible influences of OC and HOC processing alongside with the presence of protein on lipid network, the molecular arrangements of the lipid crystal networks within the developed colloidal networks were also analyzed through XRD. As shown in Figure 6a, for all the samples made with 1% RBW OG and OC, two small wide-angle diffraction peaks (as indicated by the arrows) with d-spacing of 0.41 and 0.37 nm were present. These peaks are characteristics of orthorhombic sub-cell structures of β′ lipid crystals attributed to the rice bran wax oleogels [22,59]. As expected, identical wide-angle peaks with significantly higher intensities appeared in OG and OC samples made with the 10% RBW (Figure 6b). This finding was consistent with a study by Dassanayake et al. [22] who illustrated the linear relationship between wide angle peak intensities and rice bran wax concentrations. Equivalent results were observed for the HOG and HOC samples, displaying the same β′ polymorph with d-spacing of 0.41 and 0.37 nm (Figure 6c–d). These results indicate that the presence of water in the HOG and HOC samples did not influence the polymorphic behavior of the oleogel lipid crystals. This was noteworthy given the chemical compositions of the constituents and the aforementioned hydrogen bonding interactions that may influence the lipid crystallization. Overall, the findings indicate that protein did not affect the amount of solid fat formation or the type of polymorph, suggesting that the denatured whey protein merely served as filler particles in the gel networks. 

**Figure 7:**
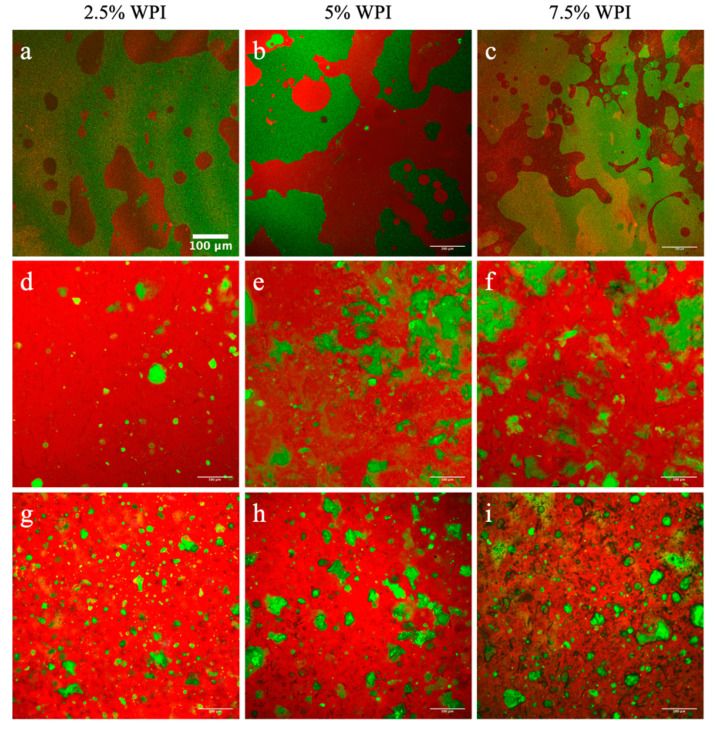
Confocal microscopy images of: (**a**–**c**) CD samples, (**d**–**f**) OC samples gelled with 1% RBW, and (**g**–**i**) OC samples gelled with 10% RBW. The lipid component is shown in red and the protein network is displayed in green.

Despite the negligible effects of whey protein on the thermal properties and oleogel lipid crystal properties within the OC and HOC matrices, whey protein may still interact with lipids and influence the overall structure of the developed gels. Hence, possible effects of colloidal interactions on the structure of OC and HOC matrices were evaluated using confocal microscopy. Specifically, the CDs appeared to consist of two separate phases of protein and liquid oil. These phases could be due to the reported thermodynamic incompatibility between whey protein and hydrophobic oil [34]. Consistent results were documented from the CDs regardless of whey protein concentration, as shown in Figure 7a–c. 

As depicted in Figure 7a–c, the CDs show an oil phase (shown in red) with the whey protein network (shown in green) distributed as large clumps of particles. When the oleogelation took place in the presence of RBW, the OC matrices displayed orderly structured networks. Unlike the CD systems (Figure 7a–c), the continuous lipid phases of the OCs made with 1% RBW (Figure 7d–f) were shown to occupy most of the matrix. Furthermore, the protein networks within the OCs showed small protein aggregates that were evenly distributed throughout the lipid phase. Most notably, the overall network appeared as a solid-like network due to the oleogelation process. Similar observations were documented for the OCs made with 10% RBW as depicted in Figure 7g–i. This finding revealed the role of the oleogelation on the structural integrity of both lipid and protein networks within the developed colloidal gels. These results agree with Bemer et al. [11] who reported that the oleogelation with the RBW in cream cheese products can create a dense network of well-defined protein and lipid globules [11]. 

To quantify the effects of adding different whey protein concentrations on the microstructure of the oleocolloid systems, mean particle area (μm^2^) as well as % filled area of the protein network were measured and reported in Table 5. As anticipated, increasing the whey protein content in the OCs led to a significantly higher protein particle area. The mean particle areas of protein networks in the 1% RBW OCs prepared with 2.5% and 7.5% (*w*/*w*) protein were 9.14 and 14.6 μm^2^, respectively (Table 5). Similarly, the 10% RBW OC samples also exhibited the same pattern where the areas of the protein networks containing 2.5% and 7.5% (*w*/*w*) protein increased from 7.68 to 19.1 μm^2^ (Table 5). These findings were consistent with previous studies that showed the whey protein denaturation resulted in formations of large whey protein aggregates that were concentration dependent [60,61]. For example, Purwanti et al. [60] reported that the whey protein aggregate size from 3% and 9% (*w*/*w*) whey protein solutions heated at 90 °C for 30 min were 49.3 and 62.7 nm, respectively [60]. In the study by Ni et al. [61], the size of whey protein aggregate at 0.1% and 5% (*w*/*v*) protein concentrations increased from 17 nm to 133 nm when heated at 85 °C for 30 min [61]. 

Taking into account that oil medium is thermodynamically unfavorable for the largely hydrophilic whey protein [33,34], the observed increase in the protein particle area clearly indicated the effects of entropically driven hydrophobic interactions (protein–protein) on the particle size of whey protein in these networks. Moreover, these results suggested that the evenly distributed protein aggregates (shown in Figure 7d–i) in the OCs interfered with the oleogel crystal lattice/arrangement. This interference could weaken the oleogel network and thus lower the start of melting (around 47.0 °C, Figure 5a–b) compared to that of the OG without protein (around 52.0–55.0 °C, Figure 4a). Although the mean particle area of protein aggregates increased with more protein, the % filled area of the protein networks in the OCs made with 10% RBW were consistent for all protein concentrations. However, it was noted that there was an increment with greater protein concentrations. The 10% RBW OCs made with 2.5% and 7.5% (*w*/*w*) WPI exhibited 9.93% and 13.3% the filled area, respectively (Table 5). This increment in the % filled area was shown to be significant for the 1% RBW OCs where the % filled areas of the samples made with 2.5% and 7.5% (*w*/*w*) protein were 5.11% and 13.2% (Table 5). 

**Figure 8:**
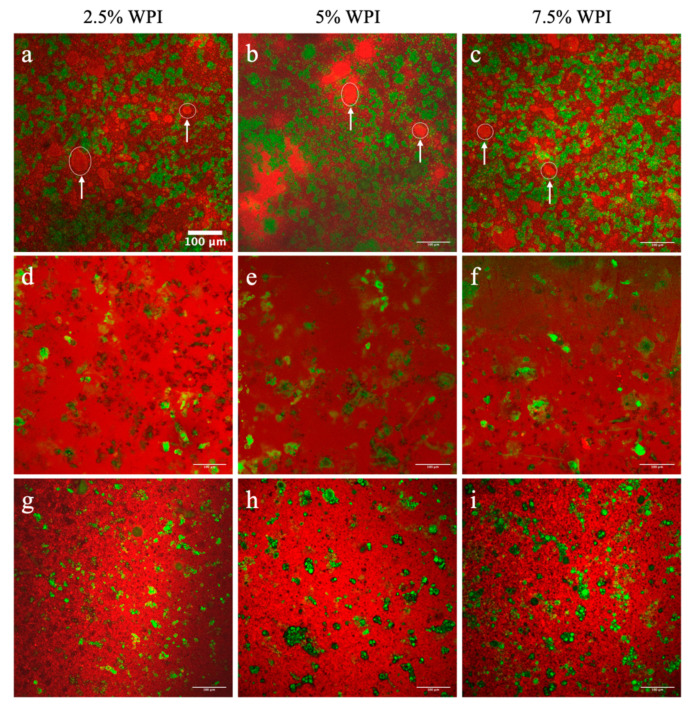
Confocal microscopy images of: (**a**–**c**) CE samples, (**d**–**f**) HOC samples gelled with 1% RBW, and (**g**–**i**) HOC samples gelled with 10% RBW. The lipid component is shown in red and the protein network is displayed in green.

Contrary to the CDs, the confocal image of CEs shown in Figure 8a–c displayed whey protein aggregates across the continuous phase. Even though the CE samples displayed a more solid-like unit compared to the CD systems (Figure 7a–c), numerous oil droplets were visible (as indicated by arrows in Figure 8a–c), representing a gel system with liquid-like nature. In parallel with the 10% RBW OCs, the % filled area of the protein components in the HOCs remained comparable regardless of protein concentrations but showed an increasing trend. 

As shown in Table 5, the % filled area of the HOCs made with 1% and 10% RBW ranged from 6.62% to 8.82% and 7.30% to 10.8%, respectively. In contrast to the OCs that showed greater particle area with more protein, the area of the protein networks in both 1% and 10% RBW HOCs (Figure 8d–i) were constant at about 5–6 μm^2^ for the given range of protein concentrations (Table 5). This constant area was associated with the entropically favored association of whey protein with water phase as well as whey protein’s strong water-holding capacity [62,63]. Assuming that the whey protein solubilized in water, then, protein–lipid interactions would be thermodynamically unfavorable due to the incompatibility between hydrophilic water and hydrophobic lipid phases. Therefore, the extent of protein–lipid interactions was thought to be greater in the OCs than the HOCs. In fact, this finding may partially help explain why the CD showed a higher denaturation temperature (102.8 °C) compared to that of the CE (73.5 °C) (Figure 1b,c) and also marginal structural changes after the OC processing (Figure 3a). 

**Figure 9:**
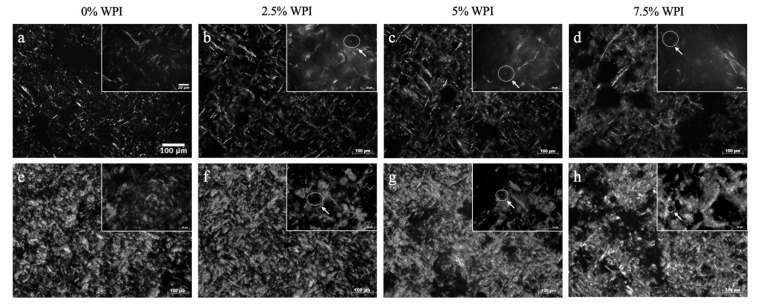
PLM images of: (**a**) OG gelled with 1% RBW, (**b**–**d**) OC samples gelled with 1% RBW, (**e**) OG gelled with 10% RBW, and (**f**–**h**) OC samples gelled with 10% RBW.

To assess how the colloidal interactions of protein in water and oil influence the oil and wax lipid networks, PLM was applied to examine structural properties of lipid crystals within the developed colloidal systems. As displayed in Figure 9a,e, the OG samples (made of 1% and 10% RBW, respectively) consisted of needle-like lipid crystals across the network. This observation is consistent with other studies that showed a needle-like lipid crystal morphology in OGs formed with RBW [22,64].

**Figure 10:**
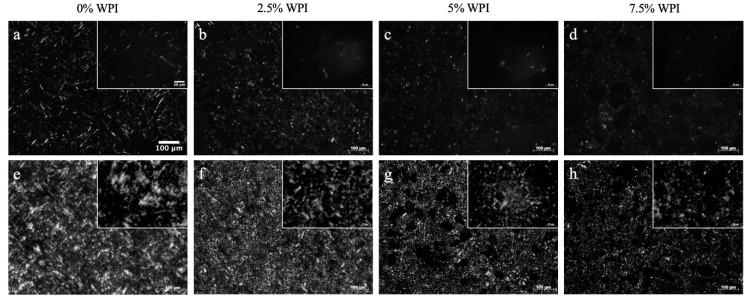
PLM images of: (**a**) HOG gelled with 1% RBW, (**b**–**d**) HOC samples gelled with 1% RBW, (**e**) HOG gelled with 10% RBW, and (**f**–**h**) HOC samples gelled with 10% RBW.

Microstructural images from PLM at the 50× magnification (shown in the in-set images of Figure 9b–d,f–h) of the OCs show that a similar lipid crystal morphology was observed even after incorporating the whey protein. In addition, globular or spherical shapes of the whey proteins were noticeably visible across the OC networks, as indicated by arrows within the in-set images. This result is similar to the work of Bolder et al. [65] who visualized a small amount of whey protein under polarized light in a pH 2 solution [65]. Moreover, as seen in Figure 9b–d,f–h, the greater amount of whey protein appeared to induce denser lipid crystals in the OC networks. The mean particle area of the lipid crystals in the 1% RBW OCs was relatively constant ranging between 15.2 and 16.4 μm^2^ whereas % fill area increased significantly from 11.2% to 15.1% with more protein content (Table 5). On the contrary, the mean particle area of the lipid crystals in the 10% RBW OCs significantly increased from 42.1 to 59.1 μm^2^ in the 2.5% and 7.5% (*w*/*w*) protein samples, respectively (Table 5). While observing an increment from 47.5% to 50.2% in the % filled area of the lipid crystals in the OCs made with 10% RBW, the increment was found to be insignificant (Table 5). Taking into account that the crystal area and the % filled area of the OG prepared with 1 and 10% RBW were 10.6 μm^2^ and 8.80% and 40.8 μm^2^ and 38.0%, respectively (Table 5), these results demonstrated that the lipid crystal networks within the OCs due to the presence of whey protein exhibited a greater density and distribution. However, these results were not consistent with ΔH of melting and SFC of the OCs, both of which decreased with increasing whey protein concentrations from 2.5% to 7.5% (*w*/*w*) (Table 4). In other words, the presence of whey protein aggregates in the OC matrix induced clustering of lipid crystals ultimately forming denser crystal networks, rather than promoting lipid crystal content. This result sounds possible considering that the whey protein networks within the OCs were not only well distributed across the lipid networks (Figure 7d–i) but also showed greater particle area with higher protein content (Table 5). While the crystal morphology of the 1 and 10% RBW HOG (shown in Figure 10a,e) was also needle-like, its particle area was 9.55 and 29.8 μm^2^, which was similar to that of the OG made with 1% RBW (10.6 μm^2^) but not with 10% RBW (40.8 μm^2^) (Table 5). 

This finding was reasonable given that the HOG contained about 2% less wax due to the presence of water in the formulations and that the aforementioned chemical interactions between water and wax crystals and polar compounds in the oil may interfere with the oleogelation process. As shown in Figure 10b–d,f–h, the lipid crystals area of the HOCs made of 1% and 10% RBW visually appeared less dense compared to their respective HOG samples (Figure 10a,e). In particular, the mean lipid crystal area of the HOCs prepared with 1% RBW ranged from 3.37–5.52 μm^2^, which was significantly lower than that of the HOG made with 1% wax (9.55 μm^2^) as reported in Table 5. Similarly, the 10% RBW HOC samples showed significantly lower lipid crystal area (8.23–11.7 μm^2^) compared to their respective HOG control sample (29.8 μm^2^) (Table 5). It is important to point out that while the mean particle area of lipid crystals in the HOCs were similar regardless of the protein content, there was a decreasing trend with greater protein content or less wax content. In other words, the crystal area of the HOC networks was proportional to the wax content. 

These results highlighted the different effects of protein–liquid medium interactions on the lipid crystal networks, as well. In case of the OCs, whey protein aggregated in oil and increased in its size as a function of protein due to the entropy related hydrophobic interactions (Figure 7d–i). This increase resulted in what appeared the clustering effects, forming denser lipid crystals (Figure 9b–d,f–h). In contrast, there was no sign of the clustering effect induced by the presence of whey protein on the lipid crystals of the HOCs. This lack of clustering was attributed to the thermodynamically favorable association between whey protein and water. As a matter of fact, the globular shapes of whey protein particles observed in the OCs (as shown in the arrows in the inset images of Figure 9) were not detected in the HOCs due to the solubilization. Instead, the area and distribution of oleogel crystals in the HOCs showed a correlation with wax content and also suggested that water could impede the oleogel growth by potentially interacting with polar lipid components, including hydroxyl groups of fatty alcohols in RBW and free fatty acids and polar compounds in oil. Overall, the obtained results highlighted the importance of the colloidal chemistry of protein in oil vs. emulsion on the structure of the OC and HOC matrices.

## 4. Conclusions

The findings from this study highlight that the whey protein behaved as filler particles that had negligible impacts on thermal properties and oleogel lipid crystal properties within the oleocolloid and hydro-oleocolloid gels. Nevertheless, the oleocolloid and hydro-oleocolloid gels showed considerably different microstructural properties. The discrepancy in their microstructures clearly illustrates a product of different oleogel and protein chemistry, which may induce different gel strength, elasticity, and so forth. Thus, a further study is warranted for an in-depth understanding of how these structural properties mediated by different solvent types (oil vs. emulsion) influence other physical properties like texture. With further investigations, oleocolloid matrices potentially hold important promises in food applications. 

## Figures and Tables

**Table 1:** Formulations of the colloidal dispersion (CD), oleogel (OG), oleocolloid (OC), colloidal emulsion (CE), hydro-oleogel (HOG), and hydro-oleocolloid (HOC) networks prepared with combinations of whey protein isolate (WPI), high oleic soybean oil (HOSO), rice bran wax (RBW), and distilled water.

Ingredient % (*w*/*w*)	Sample													
	CD			OG	OC			CE			HOG	HOC		
WPI	2.5	5	7.5	0	2.5	5	7.5	2.5	5	7.5	0	2.5	5	7.5
* HOSO	97.5	95	92.5	99	96.52	94.05	91.57	74.5	72.64	70.73	79.2	73.75	71.97	70.09
* RBW	0	0	0	1	0.975	0.95	0.925	0	0	0	0.8	0.745	0.726	0.707
Distilled Water	0	0	0	0	0	0	0	23	22.36	21.76	20.0	23.0	22.3	21.7
WPI	2.5	5	7.5	90	2.5	5	7.5	2.5	5	7.5	0	2.5	5	7.5
* HOSO	97.5	95	92.5	10	87.75	85.5	83.25	74.5	72.64	70.73	72	67.1	65.3	63.73
* RBW	0	0	0	0	9.75	9.5	9.25	0	0	0	8	7.45	7.26	7.07
Distilled Water	0	0	0	0	0	0	0	23	22.36	21.76	20.0	23.0	22.3	21.7

* The ratio of HOSO to RBW was approximately 99:1 (1% RBW) or 90:10 (10% RBW) for all colloidal gels.

**Table 2:** FTIR Analysis of the secondary structure of whey protein in CD samples before and after the OC processing.

WPI (%)	Processing	Relative % of Whey Protein Secondary Structure				
		Amino Acid Side Chain	Random Coil	β-Sheet	β-Turns	α-Helix
2.5	No	0.95 ^ab^ ± 0.1	14.4 ^a^ ± 0.8	58.2 ^b^ ± 1.2	7.84 ^a^ ± 1.0	18.6 ^c^ ± 0.6
	Yes	0.85 ^a^ ± 0.1	18.2 ^a^ ± 0.5	56.9 ^b^ ± 0.3	11.3 ^ab^ ± 0.4	13.0 ^a^ ± 0.2
5	No	0.59 ^a^ ± 0.0	17.2 ^a^ ± 1.9	55.0 ^ab^ ± 0.7	13.8 ^b^ ± 2.1	13.3 ^a^ ± 1.0
	Yes	0.71 ^a^ ± 0.2	17.2 ^a^ ± 2.4	55.0 ^ab^ ± 0.3	13.7 ^b^ ± 2.4	13.4 ^a^ ± 0.2
7.5	No	1.38 ^b^ ± 0.2	18.4 ^a^ ± 0.4	51.0 ^a^ ± 3.2	10.2 ^ab^ ± 1.1	16.2 ^b^ ± 1.2
	Yes	0.66 ^a^ ± 0.0	16.1 ^a^ ± 0.5	54.9 ^ab^ ± 0.5	8.76 ^a^ ± 0.6	19.5 ^c^ ± 0.6

Different superscripts indicate significant differences within the values of each column (*p* < 0.05).

**Table 3:** FTIR Analysis of the secondary structure of whey protein in CE samples before and after the HOC processing.

WPI (%)	Processing	Relative % of Whey Protein Secondary Structure				
		Amino Acid Side Chain	Random Coil	β-Sheet	β-Turns	α-Helix
2.5	No	2.82 ^ab^ ± 0.3	10.4 ^a^ ± 1.5	51.5 ^a^ ± 2.5	15.4 ^c^ ± 0.4	19.9 ^d^ ± 0.9
	Yes	1.90 ^a^ ± 0.3	17.0 ^b^ ± 0.7	57.5 ^b^ ± 0.7	11.5 ^bc^ ± 0.5	12.0 ^b^ ± 0.3
5	No	0.89 ^a^ ± 0.2	15.1 ^ab^ ± 2.3	49.7 ^a^ ± 2.5	15.0 ^c^ ± 2.5	17.5 ^cd^ ± 1.4
	Yes	2.13 ^a^ ± 0.1	15.2 ^ab^ ± 1.8	65.8 ^c^ ± 0.2	6.71 ^a^ ± 1.4	9.40 ^a^ ± 0.8
7.5	No	4.59 ^b^ ± 2.0	16.5 ^ab^ ± 0.0	48.8 ^a^ ± 1.5	11.2 ^bc^ ± 2.0	17.5 ^c^ ± 0.3
	Yes	1.85 ^a^ ± 0.3	18.6 ^b^ ± 3.1	57.6 ^b^ ± 1.2	10.1 ^ab^ ± 2.0	11.8 ^b^ ± 0.7

Different superscripts indicate significant differences within the values of each column (*p* < 0.05).

**Table 4:** Thermal properties [onset of melting (OM), melting point (MP), enthalpy (ΔH)], and solid fat content (SFC and normalized * SFC) of the colloidal networks.

	**1% RBW Samples**							
	**OG**	**OC**			**HOG**	**HOC**		
WPI (%)	0	2.5	5	7.5	0	2.5	5	7.5
OM (°C)	55.3 ^b^ ± 0.4	47.1 ^a^ ± 0.3	47.5 ^a^ ± 0.5	48.0 ^a^ ± 1.0	47.4 ^a^ ± 0.6	53.2 ^b^ ± 0.7	53.5 ^b^ ± 1.4	53.9 ^b^ ± 1.0
MP (°C)	61.9 ^a^ ± 0.7	62.1 ^a^ ± 0.8	62.5 ^a^ ± 0.9	62.2 ^a^ ± 0.4	62.2 ^a^ ± 0.9	62.0 ^a^ ± 0.2	63.0 ^a^ ± 2.1	62.8 ^a^ ± 1.6
ΔH (J/g)	0.61 ^d^ ± 0.0	0.65 ^d^ ± 0.0	0.51 ^cd^ ± 0.1	0.38 ^ac^ ± 0.1	0.60 ^bd^ ± 0.0	0.41 ^bc^ ± 0.1	0.27 ^ab^ ± 0.0	0.21 ^a^ ± 0.1
SFC (%)	0.76 ^e^ ± 0.0	0.73 ^de^ ± 0.1	0.67 ^cd^ ± 0.1	0.63 ^c^ ± 0.0	0.60 ^c^ ± 0.1	0.59 ^bc^ ± 0.1	0.52 ^ab^ ± 0.1	0.47 ^a^ ± 0.1
* SFC (%)	0.76 ^b^ ± 0.0	0.75 ^b^ ± 0.1	0.70 ^ab^ ± 0.1	0.69 ^ab^ ± 0.0	0.75 ^b^ ± 0.1	0.75 ^b^ ± 0.1	0.72 ^ab^ ± 0.1	0.64 ^a^ ± 0.0
	**10% RBW Samples**							
	**OG**	**OC**			**HOG**	**HOC**		
WPI (%)	0	2.5	5	7.5	0	2.5	5	7.5
OM (°C)	52.5 ^B^ ± 1.4	47.1 ^A^ ± 1.7	47.7 ^A^± 1.2	47.0 ^A^ ± 2.1	46.8 ^A^ ± 0.8	51.0 ^AB^ ± 2.4	50.6 ^AB^ ± 1.5	50.5 ^AB^ ± 0.6
MP (°C)	71.8 ^AB^ ± 0.6	73.1 ^B^ ± 0.1	72.2 ^AB^ ± 0.3	72.3 ^AB^ ± 1.0	72.2 ^A,B^ ± 0.6	71.6 ^AB^ ± 0.2	71.6 ^AB^ ± 0.3	70.5 ^A^ ± 1.1
ΔH (J/g)	19.8 ^C^ ± 0.4	20.1 ^C^ ± 0.4	20.4 ^C^ ± 1.3	17.1 ^B^ ± 0.7	20.1 ^BC^ ± 1.6	18.6 ^BC^ ± 0.8	17.1 ^B^ ± 1.1	12.7 ^A^ ± 0.3
SFC (%)	8.75 ^D^ ± 0.3	8.35 ^CD^ ± 0.3	8.63 ^D^ ± 0.5	7.82 ^C^ ± 0.4	6.96 ^B^ ± 0.1	6.44 ^B^ ± 0.5	6.37 ^B^ ± 0.4	5.13 ^A^ ± 0.6
* SFC (%)	8.75 ^B^ ± 0.3	8.56 ^B^ ± 0.3	9.08 ^B^ ± 0.5	8.45 ^B^ ± 0.5	8.70 ^B^ ± 0.1	8.64 ^B^ ± 0.6	8.78 ^B^ ± 0.5	7.25 ^A^ ± 0.8

Superscripts show significant differences within the values in each row (*p* < 0.05).

**Table 5:** Particle area and percent filled area of protein and lipid crystal networks within the colloidal networks, obtained from confocal and PLM micrographs.

	**1% RBW Samples**							
	**OG**	**OC**			**HOG**	**HOC**		
WPI (%)	0	2.5	5	7.5	0	2.5	5	7.5
Protein Particle Area (μm^2^)	NA *	9.14 ^ab^ ± 0.6	11.9 ^bc^ ± 2.8	14.6 ^c^ ± 2.5	NA *	6.73 ^a^ ± 0.7	5.88 ^a^ ± 1.2	6.33 ^a^ ± 1.6
Protein % Filled Area	NA*	5.11 ^a^ ± 0.5	11.0 ^bc^ ± 2.5	13.2 ^c^ ± 3.5	NA *	6.62 ^ab^ ± 0.4	8.74 ^abc^ ± 2.4	8.82 ^abc^ ± 1.6
Lipid Crystals Particle Area (μm^2^)	10.6 ^b^ ± 1.4	15.2 ^c^ ± 1.8	16.4 ^c^ ± 1.4	16.0 ^c^ ± 2.1	9.55 ^b^ ± 2.0	5.52 ^a^ ± 0.5	4.28 ^a^ ± 1.2	3.37 ^a^ ± 0.9
Lipid Crystals % Filled Area	8.80 ^cd^ ± 0.9	11.2 ^de^ ± 2.9	12.4 ^ef^ ± 3.5	15.1 ^f^ ± 2.6	5.88 ^bc^ ± 1.4	3.37 ^ab^ ± 0.6	2.63 ^a^ ± 0.5	1.73 ^a^ ± 0.5
	**10% RBW Samples**							
	**OG**	**OC**			**HOG**	**HOC**		
WPI (%)	0	2.5	5	7.5	0	2.5	5	7.5
Protein Particle Area (μm^2^)	NA *	7.68 ^AB^ ± 2.2	11.0 ^B^ ± 2.2	19.1 ^C^ ± 1.6	NA *	5.51 ^A^ ± 0.4	5.49 ^A^ ± 0.9	4.80 ^A^ ± 0.9
Protein Network % Filled Area	NA *	9.93 ^A^ ± 2.7	11.1 ^A^ ± 3.2	13.3 ^A^ ± 3.0	NA *	7.30 ^A^ ± 0.9	8.30 ^A^ ± 2.6	10.8 ^A^ ± 1.8
Lipid Crystals Particle Area (μm^2^)	40.8 ^C^ ± 6.8	42.1 ^C^ ± 3.6	46.6 ^C^ ± 4.4	59.1 ^D^ ± 2.7	29.8 ^B^ ± 4.8	11.7 ^A^ ± 2.6	11.4 ^A^ ± 2.1	8.23 ^A^ ± 1.8
Lipid Crystals % Filled Area	38.0 ^B^ ± 9.4	47.5 ^C^ ± 1.7	49.6 ^C^ ± 3.6	50.2 ^C^ ± 6.1	37.0 ^B^ ± 5.0	23.1 ^A^ ± 2.1	22.7 ^A^ ± 3.4	20.6 ^A^ ± 4.5

* Not applicable for the OG and HOG (the control group without protein). Superscripts show significant differences within the values in each row (*p* < 0.05).
